# The network of microRNAs, transcription factors, target genes and host genes in human renal cell carcinoma

**DOI:** 10.3892/ol.2014.2683

**Published:** 2014-11-07

**Authors:** CHENGLU SONG, ZHIWEN XU, YUE JIN, MINGHUI ZHU, KUNHAO WANG, NING WANG

**Affiliations:** 1 Department of Computer Science and Technology, Jilin University, Changchun, Jilin 130012, P.R. China; 2 Key Laboratory of Symbol Computation and Knowledge Engineering, Ministry of Education, Jilin University, Changchun, Jilin 130012, P.R. China

**Keywords:** transcription factors, microRNA, target genes, host genes, network, renal cell carcinoma

## Abstract

At present, scientists have performed numerous studies investigating the morbidity of renal cell carcinoma (RCC) in the genetic and microRNA (miRNA) fields, obtaining a substantial amount of knowledge. However, the experimentally validated data of genes, miRNA and transcription factors (TFs) cannot be found in a unified form, which makes it challenging to decipher the regulatory mechanisms. In the present study, the genes, miRNAs and TFs involved in RCC are regarded as elements in the regulatory network, and the present study therefore focuses on the association between each entity. Three regulatory networks were constructed hierarchically to indicate the regulatory association between the genes, miRNAs and TFs clearly, including the differentially expressed, associated and global networks. All the elements were macroscopically investigated in these networks, instead of only investigating one or several of them. The present study not only compared and analyzed the similarities and the differences between the three networks, but also systematically expounded the pathogenesis of RCC and supplied theoretical foundations for future gene therapy investigations. Following the construction of the three networks, certain important pathways were highlighted. The upstream and downstream element table of differentially expressed genes and miRNAs was listed, in which self-adaption associations and circle-regulations were identified. In future studies, the identified genes and miRNAs should be granted more attention.

## Introduction

Renal cell carcinoma (RCC) is a kidney cancer that derives from the lining of the proximal convoluted tubule and the small tubes in the kidney that filter the blood to remove waste products. RCC is the most common type of kidney cancer in adults, responsible for ~80% of cases, and is also known to be the most lethal of all the genitourinary tumors (1).

Although an increasing number of individuals succumb to RCC and studies have begun to focus on the differentially expressed genes and microRNAs (miRNAs) in RCC, the signals and mechanisms that govern miRNA transcriptional regulation remain unclear. Experimental data indicates that differentially expressed genes and differentially expressed miRNAs play key roles in the development, metastasis and therapy of RCC. For example, polymorphisms in vascular endothelial growth factor are associated with an increased risk of developing RCC (2). The tumor suppressor gene DKK1 induces apoptosis and inhibits proliferation in human RCC cells (3). The majority of regulatory genes encode transcription factors (TFs), which modulate gene expression by binding the regulatory sequences of their target genes. TFs and miRNAs are prominent regulators of gene expression (4). TFs are proteins that can activate or repress transcription by binding *cis*-regulatory elements, located in the upstream regions of genes and regulate gene expression at the transcriptional level, either individually or joint with other proteins.

MiRNAs can be located within numerous genes and are named for their host genes. Rodriguez *et al* indicated that miRNAs were transcribed in parallel with their host transcripts and two different transcription classes of miRNAs, exonic and intronic, were identified that may require slightly different mechanisms of biogenesis (5). Baskerville and Bartel indicated that intronic miRNAs and their host genes exhibited a close association (6). Intronic miRNAs and their host genes are usually coordinately expressed in biological progression. They usually act as potential partners to achieve biological function and also affect the alteration of pathways (7). All results suggest that miRNAs can work together with their host genes or separately to contribute to the progression of cancer. In the present study, when the miRNA is differently expressed the host genes are considered to also be differently expressed and involved in the progression of cancer.

The present study aimed to extract the associations between genes, miRNAs and their host genes, and these transcriptional associations were considered to be a point of penetration to build the regulatory network of the genes and miRNAs involved in RCC. Three levels of networks were obtained, the differentially expressed, associated and global networks. The global network contained all the experimentally validated pathways for the genes and miRNA. However, this network was so complex that the pathways associated with RCC could not be easily identified. Therefore, the other two networks were used for further research. Pathways for the differentially expressed elements were extracted separately and the differentially expressed network partially uncovered how RCC forms. The topology network was found in the progression of RCC. The similarities and differences between the three networks were compared and analyzed to distinguish the key nodes and pathways. The network of differentially expressed elements partially uncovered the mechanism of RCC.

## Materials and methods

### Material collection and data processing

Target miRNA interactions were extracted from two databases, TarBase 5.0 (http://diana.cslab.ece.ntua.gr/tarbase/) and miRTarBase (http://mirtarbase.mbc.nctu.edu.tw/). TarBase 5.0 is a comprehensive database with experimentally supported animal miRNA targets and miRTarBase is a database of experimentally validated miRNA-targets interactions. Different databases use different symbols to represent miRNAs and genes. In order to unify the method of symbolic representation, the official symbols from the National Center for Biotechnology Information (NCBI) database, which can be accessed online (www.ncbi.nlm.nih.gov/gene/), were used. These experimentally validated data strongly support the present study. This dataset was considered to be set *U*_1_.

The TF-miRNA interactions were extracted from TransmiR (http://cmbi.bjmu.edu.cn/transmir) (8). The data on TransmiR were extracted from the public literature and biological experiments. This dataset was considered to be set *U*_2_.

Information on the miRNA and host genes was extracted from miRBase (http://www.mirbase.org/) and NCBI. MiRBase provides a collection of all the confirmed human miRNAs. The official symbols and official IDs from NCBI were used to indicate host genes and their miRNAs. This dataset was considered to be set *U_3_.*

The differentially expressed genes of RCC were mainly extracted from the KEGG pathway database and Cancer Genetics Web, which can be accessed online (www.cancerindex.org). The KEGG pathway database (www.kegg.com/kegg/pathway.html) consists of graphical diagrams of biochemical pathways and certain known regulatory pathways. The RCC pathway map was obtained from this database. The map demonstrates all the validated mutated RCC genes. Similar methods were used to extract the mutated RCC genes from Cancer Genetics Web (accessed online at www.cancer-genetics.org). To complete the data collection, studies on the mutated genes of RCC were manually searched for using the Science Citation Index (SCI; Thomson-Reuters Corporation, New York, NY, USA). The RCC associated genes include the differentially expressed genes of RCC and other associated genes for which the pertinent literature was manually searched. In the present study, differentially expressed genes were considered to be a part of the associated genes in the RCC-associated network. Additionally, TFs that may be involved in RCC were extracted using the P-match method. P-Match is a novel tool to identify TF binding sites in DNA sequences. The tool combines pattern matching with weight matrix approaches, and thus provides higher accuracy of recognition than each of the methods alone. TFs were considered to be RCC-associated genes and only the TFs in set *U*_2_ were focused on. Promoter region sequences, 1,000 and 5,000 nt in length, of targets targeted by mutated miRNAs were downloaded from the University of California Santa Cruz database (http://genome.ucsc.edu/). The P-match method was used to identify the TF binding sites in the 1000 and 5000 nt promoter region sequences and mapped TFBSs onto the promoter region of targets. The matrix library of P-Match is contains sets of known TF-binding sites collected in TRANSFAC, and therefore provides the possibility of searching for a large variety of TF binding sites. The complete data of differentially expressed genes and associated genes were considered to be data set *U*_4_.

Differentially expressed miRNAs were extracted from mir2Disease (9). Mir2Disease is a manually curated database that aims to provide a comprehensive resource of miRNA deregulation in various human diseases. To complete the data collection, studies on RCC were searched for using SCI. The complete differentially expressed miRNAs and associated miRNAs of RCC are considered as set *U*_5_.

### Three level networks construction

The transcriptional network of RCC is an extremely complex regulatory network. Differentially expressed genes and miRNAs play key roles in this network. They participate in cancer progression, including carcinogenesis, metastasis and therapy. Therefore, the core network in RCC was extracted using the following method: Differentially expressed data from *U*_4_ and *U*_5_ was mapped onto *U*_1_, *U*_2_ and *U*_3,_ and then the regulatory associations of TF-miRNA, miRNA-targets and host gene-miRNA were extracted. Following the combination of all the associations, the core network was obtained.

In addition to differentially expressed genes and miRNAs, the RCC-associated genes and miRNAs also influence the key cellular processes of RCC. Therefore, the network of RCC-associated elements was used to further illuminate the regulatory network of RCC. Naturally, this network includes the core network and it has more complex regulatory associations compared with the core network. The regulatory network in RCC was extracted using the aforementioned method.

The former two networks present extremely important regulatory associations in RCC. In addition to the experimentally validated genes and miRNAs that are included in the former two networks, certain genes and miRNAs that are not experimentally validated may be involved in the progression of RCC. In the third network, the complete TF and miRNA interaction that were present in the associated network were mapped onto *U*_1_, *U*_2_ and *U*_3_, and then the regulatory associations of TF-miRNA, miRNA-targets and host gene-miRNA were extracted. Following the combination of all the associations, the expanded global network was obtained.

## Results and Discussion

### Core transcriptional network of RCC

Through statistical analysis, a core transcriptional network that attempts to describe the mechanism of human RCC was obtained. There were two TFs, phosphatase and tensin homolog (PTEN) and tumor protein p53 (TP53) in this network, which were regarded as the essential regulatory elements.

**Figure 1:**
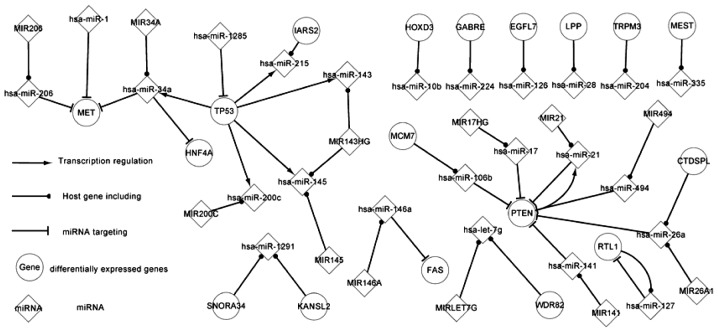
Core transcriptional network of differentially expressed genes, miRNAs and host genes in renal cell carcinoma. miRNA, microRNA

Fig. 1 shows the core transcriptional network, consisting of few regulatory pathways. PTEN regulates hsa-miR-21 and TP53 regulates five miRNAs, consisting of hsa-miR-143, hsa-miR-145, hsa-miR-200, hsa-miR-215 and hsa-miR-34a. These five miRNAs target two genes, HNF4A and MET. These genes and miRNAs are extremely important in the progression of RCC. Wirsing *et al* indicated that miR-34a overexpression in RCC cooperates with the downregulation of HNF4A mRNA (10).

The product of the TP53 gene is tumor protein p53. This protein acts as a tumor suppressor and regulates cell division by preventing cells from growing and dividing too rapidly or in an uncontrolled way (11). Mutations in the TP53 gene may aid predictions of whether the RCC will progress and spread to nearby tissues and whether the disease will recur following treatment.

The tumor suppressor gene PTEN is mutated or homozygously deleted in numerous cancers and maps to a region of 10q within the reported region of minimal loss in RCC (12). PTEN participates in two overlaps and targets hsa-miR-106b, hsa-miR-141, hsa-miR-17, hsa-miR-21, hsa-miR-26a and hsa-miR-494 whilst simultaneously being targeted by them.

The present study attempted to use the core transcriptional network to describe the mechanism of RCC. In the following section, the more complex regulatory network will be discussed.

### RCC-associated network

**Figure 2:**
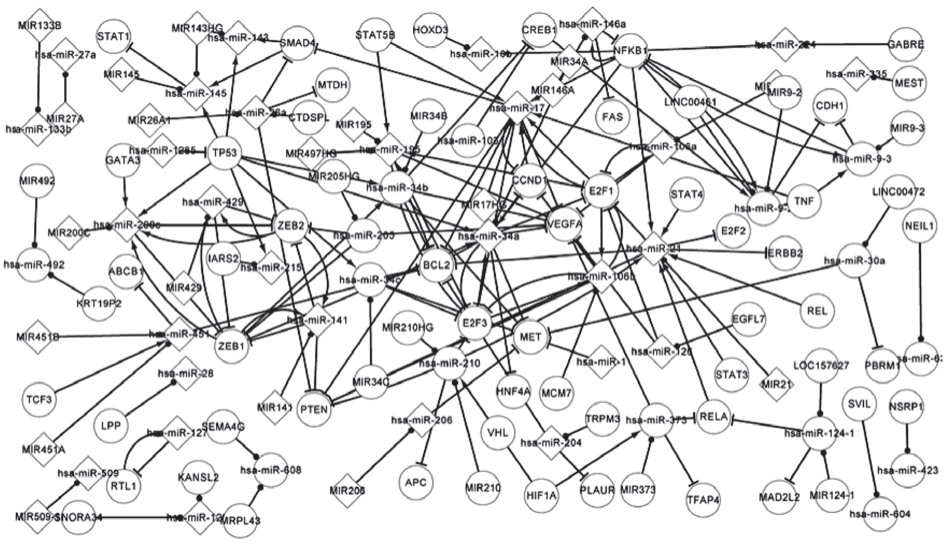
Renal cell carcinoma-associated network consisting of the relevant genes, microRNAs and host genes in renal cell carcinoma.

The associated regulatory network of RCC consists of differentially expressed genes and miRNAs, associated genes and miRNAs, targets of miRNAs and host genes of miRNAs. Naturally, the associated network includes the core network. Fig. 2 shows more complex regulatory associations than Fig. 1.

Fig. 1 shows that there are two TFs, PTEN and TP53, in the regulatory network. Therefore, these two TFs were regarded as the essential regulatory elements. In Fig. 2, there are 20 TFs in the regulatory network. These 20 TFs include the two essential TFs presented in Fig. 1 and regulate 22 mutated miRNAs. The TFs in the network, TP53, ZEB1 and NFKB1, regulate more miRNA expression and have a high potential for being more influential with regard to the overall behavior of the network compared with others (13,14). NFKB1 regulates hsa-miR-146a expression, and hsa-miR-146a targets FAS and NFKB1 itself. Therefore, NFKB1 and hsa-miR-146a constitute a feedback loop.

Fig. 2 shows additional pathways that affect the progression of RCC compared with Fig. 1. In order to briefly explain this, only certain additional pathways for differentially expressed TFs and additional miRNAs are described. ZEB1 encodes a zinc finger TF. The encoded protein likely plays a role in the transcriptional repression of interleukin 2 (15). Through this picture it can be predicted that mutations in this gene may associate with RCC. ZEB1 regulates hsa-miR-141, hsa-miR-200c, hsa-miR-34a, hsa-miR-34b and hsa-miR-429 expression. Liu *et al* identified that miR-200c microRNAs and E-cadherin maintain a higher level of expression by repressing ZEB1 (16). These five miRNAs target 12 relevant genes, including PTEN, MET and CCND1. It has been demonstrated that an ESR1-mediated decrease in hsa-miR-21 expression correlates with increased protein expression of endogenous hsa-miR-21 targets, including PDCD4, PTEN and BCL2 (17).

NFKB1 is another important TF that regulates the expression of eight miRNAs, including hsa-miR-21, hsa-miR-17 and hsa-miR-224. In the present network, a total of 16 feedback loops, consisting of seven genes and 12 miRNAs, were identified. Two differentially expressed genes were involved in the feedback loops.

### Global network of RCC

The global regulatory network shows additional comprehensive regulatory associations of RCC, including all the associations in *U*_1_, *U*_2_ and *U*_3_, and contains a larger number of TFs, targets, miRNAs and miRNA host genes compared with the associated network. The global regulatory network also includes the differentially expressed and associated networks.

### Comparison and analysis of the genetic role of differentially expressed genes

Nodes were classed according to the regulatory association of adjacent nodes in the three network levels for comparing and analyzing the interacting features of each differentially expressed gene. Among these genes, two genes, PTEN and TP53, demonstrated the particular feature of regulating miRNA and being targeted by the miRNA.

Initially, the present study focused on the genes. The first class of gene possesses six types of adjacent nodes, consisting of three types of successors and three types of predecessors. This class of gene includes PTEN and TP53. Table I shows PTEN, predecessors of PTEN and successors of PTEN as well as their regulatory associations.

Table I shows six miRNAs that target PTEN. In the core, associated and global networks, PTEN regulates one, one and 10 miRNAs, respectively. Additionally, in the associated and global networks, PTEN is targeted by five and 22 miRNAs, respectively. These predecessors indirectly affect successors through PTEN. Among all the miRNAs, it was found that hsa-miR-21 targets and is regulated by PTEN in the three networks. PTEN and hsa-miR-21 form a self-adaption association. They are each differentially expressed elements in RCC. Therefore, PTEN and hsa-miR-21 must play a key role in the progression of RCC. Dey *et al* revealed that miR-21 targets the PTEN mRNA 3′ untranslated region to decrease PTEN protein expression and augments Akt phosphorylation in renal cancer cells, and also revealed that downregulation of PTEN and overexpression of constitutively active Akt kinase prevented miR-21 Sponge-induced inhibition of renal cancer cell proliferation and migration (18).

Secondly, the remaining genes that do not regulate any miRNA were focused on. The first class of gene possesses three types of adjacent nodes, consisting of three types of predecessors, including APC, FAS, HNF4A and MET. They are only targeted by certain miRNAs, but do not regulate any miRNA. It was suggested that these may be the last nodes in the pathway.

The second class of gene possesses one type of adjacent node, a type of predecessor, such as GRB2. GRB2 is targeted by two miRNAs in the global network and it does not regulate any miRNA. It was suggested that GRB2 has the least effect compared with other differentially expressed genes.

The third class of gene possesses two types of adjacent nodes, two types of successors or two types of predecessors, including PBRM1 and VHL. PBRM1 has predecessor in the associated network and global network, and it does not regulate any miRNA. VHL possesses successors in the associated and global networks and it does not target any miRNA.

The final class of gene only possesses a type of adjacent node, such as HSPA1B or IL6. There are also certain genes that possess no adjacent nodes, including CA9 and FH, that are not in the present discussion.

### Comparison and analysis of the features of differentially expressed miRNAs

Similar to analyzing differentially expressed genes, the same method was used to analyze 38 differentially expressed miRNAs. In Table II, hsa-miR-34a was set as an example and the precursors and successors of hsa-miR-34a in the differentially expressed, associated and global networks are listed. hsa-miR-34a possesses six types of adjacent nodes, three types of predecessors and three types of successors.

In the differentially-expressed network, TP53 regulates hsa-miR-34a and hsa-miR-34a targets HNF4A and MET. In the associated network, E2F3, NFKB1, TP53 and ZEB1 regulates has-miR-34a, while hsa-miR-34a targets E2F1, E2F3, HNF4A, MET, CCND1, BCL2 and VEGFA. In the associated network, there is a specific gene, E2F3, which forms a self-adaption association with hsa-miR-34a.

The method that was used for the differentially expressed genes was used to compare and analyze each differentially expressed miRNA. Among the present miRNAs, there were 38 differentially expressed miRNAs. The first class of miRNA possessed six types of adjacent nodes, three types of predecessors and three types of successors, including hsa-miR-143. The second class of miRNA possessed five types of adjacent nodes, including hsa-miR-141. Hsa-miR-141 is targeted by three genes and is regulated by two genes. The classification method used for miRNAs was similar to that used for the genes and is therefore not explained.

### Comparison and analysis of the features of popular TFs

The same method that was used for the genes and miRNA was used to compare and analyze each popular TF in the associated network. Numerous TFs, including CREB1, E2F1, NFKB1 and ZEB1, and the corresponding miRNAs were found to form self-adaption associations. Table III uses ZEB1 as an example.

In the differentially expressed network, ZEB1 is not targeted by any miRNAs and does not regulate any miRNAs. In the associated network, ZEB1 regulates hsa-miR-141, hsa-miR-200c, hsa-miR-34a, hsa-miR-34b, hsa-miR-429 and hsa-miR-141. Hsa-miR-200c, hsa-miR-205 and hsa-miR-429 target ZEB1. ZEB1 forms self-adaption associations with hsa-miR-141, hsa-miR-200c and hsa-miR-429.

### Analysis of host genes and miRNA in RCC

Host genes and their miRNA demonstrate certain important features in the present study. Although these host genes are not differentially expressed in RCC, they are considered to be differentially expressed genes when the miRNAs is differentially expressed. Fig. 1 shows certain host gene and miRNA pathways. For example, the MCM7 gene codes for hsa-miR-106b, which targets PTEN. There is a notable association between hsa-miR-21 and PTEN. The PTEN gene includes hsa-miR-21 and is also targeted by hsa-miR-21. A host gene includes several miRNAs. For example, the MIR143HG gene includes hsa-miR-143 and hsa-miR-145. One miRNA can also be located in several genes. For example, two host genes, KANSL2 and SNORA34, include hsa-miR-1291. It was suggested that host genes and their miRNAs may aid the understanding of the pathogenesis of RCC.

### Transcriptional network of TFs and differentially expressed miRNAs

**Figure 3:**
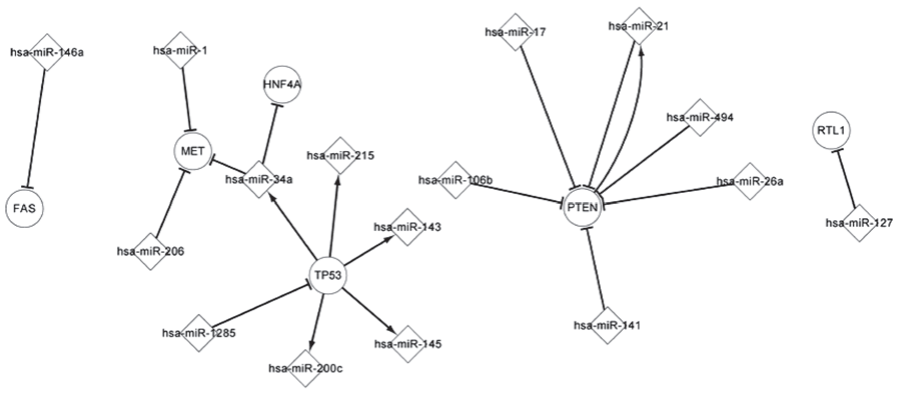
Specific host genes and their regulatory associations with transcription factors, targets and microRNAs.

Fig. 3 shows the transcriptional network of popular TFs and differentially expressed miRNAs. There are 32 different miRNAs in the differentially expressed network. In total, 16 differentially expressed miRNAs are included in Fig. 3. These miRNAs and popular TFs construct a transcriptional network, which presents several significant characters in the progression of RCC. TFs and miRNAs exhibit several types of regulatory associations that precisely affect the expression of their targeted elements. Fig. 3 shows that PTEN regulates one miRNA and is targeted by six miRNAs. The five miRNAs, hsa-miR-143, hsa-miR-145, hsa-miR-200c, hsa-miR-215 and hsa-miR-34a, are regulated by TP53 and can target HNF4A and MET. PTEN regulates and is targeted by hsa-miR-21. The aforementioned and differentially expressed miRNAs interact with each other to affect the progression of RCC. Fig. 3 also shows that one differentially expressed miRNA can be regulated by several TFs and can indirectly affect another miRNA through TFs, and Fig. 3 also shows that one TF can be targeted by several differentially expressed miRNAs and indirectly affect another TF through differentially expressed miRNA.

### Conclusion

All the currently validated genes and miRNAs associated with RCC were collected in the present study and three regulatory networks were used to analyze the complex regulatory associations of the differentially expressed elements in RCC. The present study extracted and compared the similarities and differences of all the differentially expressed elements in the three networks to distinguish the key nodes and pathways that contribute to understanding the mechanism of the carcinogenicity and the therapy of RCC. It was found that certain pathways of differentially expressed elements have been validated in RCC and other pathways that have not been validated in RCC affect other cancers. Certain associated pathways in the associated network and in additional pathways in the global network were also found to affect the progression of other cancers. Pathways of differentially expressed elements must be involved in RCC, but the majority of these mechanisms remain unclear. The pathways that are not validated in RCC can affect the progression of cancer whether they play similar or novel roles in RCC. Additional research on these pathways in RCC is required. The present study supplied comprehensive data associated with RCC that will guide medical investigators and biologists to further achieve pertinent research about the mechanisms of differentially expressed genes and miRNAs in RCC. In future studies, transcription co-factors and the interaction between proteins may be considered in the present network, which may derive a more comprehensive and extensive network for RCC. In-depth research into the pathogenesis and treatment of RCC using such ample data may also be a focus of future studies.

## Figures and Tables

**Table I:** Regulatory associations between miRNAs and PTEN.

miRNAs that target gene				miRNA that is regulated by gene		
		
Differentially expressed miRNAs	Associated miRNAs	Global miRNAs	Gene	Differentially expressed miRNAs	Associated miRNAs	Global miRNAs
hsa-miR-106b	hsa-miR-106b	hsa-miR-106b	PTEN	hsa-miR-21	hsa-miR-21	hsa-miR-19a
hsa-miR-141	hsa-miR-141	hsa-miR-141	PTEN	0	0	hsa-miR-21
hsa-miR-17	hsa-miR-17	hsa-miR-17	PTEN	0	0	hsa-miR-22
hsa-miR-21	hsa-miR-21	hsa-miR-18a	PTEN	0	0	hsa-miR-25
hsa-miR-26a	hsa-miR-26a	hsa-miR-19a	PTEN	0	0	hsa-miR-302
hsa-miR-494	0	hsa-miR-19b	PTEN	0	0	hsa-miR-302a
0	0	hsa-miR-19b-1	PTEN	0	0	hsa-miR-302b
0	0	hsa-miR-19b-2	PTEN	0	0	hsa-miR-302c
0	0	hsa-miR-20	PTEN	0	0	hsa-miR-302d
0	0	hsa-miR-20a	PTEN	0	0	hsa-miR-302f
0	0	hsa-miR-21	PTEN	0	0	0
0	0	hsa-miR-214	PTEN	0	0	0
0	0	hsa-miR-216	PTEN	0	0	0
0	0	hsa-miR-216a	PTEN	0	0	0
0	0	hsa-miR-217	PTEN	0	0	0
0	0	hsa-miR-221	PTEN	0	0	0
0	0	hsa-miR-222	PTEN	0	0	0
0	0	hsa-miR-26a	PTEN	0	0	0
0	0	hsa-miR-26a-1	PTEN	0	0	0
0	0	hsa-miR-26a-2	PTEN	0	0	0
0	0	hsa-miR-494	PTEN	0	0	0
0	0	hsa-miR-91	PTEN	0	0	0

miRNA, microRNA.

**Table II:** Partially shows regulatory relations between hsa-miR-143 and genes in three networks.

Genes that regulate miRNA				Target genes of miRNA		
		
Differentially expressed network	Associated network	Global network	miRNA	Differentially expressed network	Associated network	Global network
TP53	E2F3	CEBPA	hsa-miR-34a	HNF4A	E2F1	AXIN2
0	NFKB1	E2F3	hsa-miR-34a	MET	E2F3	BCL2
0	TP53	MYC	hsa-miR-34a	0	HNF4A	BIRC3
0	ZEB1	NFKB1	hsa-miR-34a	0	MET	CCND1
0	0	NR1H4	hsa-miR-34a	0	CCND1	CCND3
0	0	SNAI1	hsa-miR-34a	0	BCL2	CCNE2
0	0	TP53	hsa-miR-34a	0	VEGFA	CD44
0	0	ZEB1	hsa-miR-34a	0	0	CDC25A
0	0	0	hsa-miR-34a	0	0	CDC25C
0	0	0	hsa-miR-34a	0	0	CDK4
0	0	0	hsa-miR-34a	0	0	CDK6
0	0	0	hsa-miR-34a	0	0	CEBP

miRNA, microRNA.

**Table III:** Regulatory relation between miRNAs and ZEB1.

miRNA that targets gene				miRNA that is regulated by gene		
		
Differentially expressed miRNAs	Associated miRNAs	Global miRNAs	Gene	Differentially expressed miRNAs	Associated miRNAs	Global miRNAs
0	hsa-miR-141	hsa-miR-141	ZEB1	0	hsa-miR-141	hsa-let-7
0	hsa-miR-200c	hsa-miR-200a	ZEB1	0	hsa-miR-200c	hsa-let-7a
0	hsa-miR-205	hsa-miR-200b	ZEB1	0	hsa-miR-34a	hsa-let-7a-1
0	hsa-miR-429	hsa-miR-200c	ZEB1	0	hsa-miR-34b	hsa-let-7a-2
0	0	hsa-miR-205	ZEB1	0	hsa-miR-429	hsa-let-7a-3
0	0	hsa-miR-429	ZEB1	0	0	hsa-let-7b
0	0	0	ZEB1	0	0	hsa-let-7c
0	0	0	ZEB1	0	0	hsa-let-7d
0	0	0	ZEB1	0	0	hsa-let-7e
0	0	0	ZEB1	0	0	hsa-let-7f
0	0	0	ZEB1	0	0	hsa-let-7f-1
0	0	0	ZEB1	0	0	hsa-let-7f-2
0	0	0	ZEB1	0	0	hsa-let-7g
0	0	0	ZEB1	0	0	hsa-let-7i
0	0	0	ZEB1	0	0	hsa-miR-141
0	0	0	ZEB1	0	0	hsa-miR-200a
0	0	0	ZEB1	0	0	hsa-miR-200b
0	0	0	ZEB1	0	0	hsa-miR-200c
0	0	0	ZEB1	0	0	hsa-miR-34
0	0	0	ZEB1	0	0	hsa-miR-34a
0	0	0	ZEB1	0	0	hsa-miR-34b
0	0	0	ZEB1	0	0	hsa-miR-429
